# Genetically Guided Statin Therapy on Statin Perceptions, Adherence, and Cholesterol Lowering: A Pilot Implementation Study in Primary Care Patients

**DOI:** 10.3390/jpm4020147

**Published:** 2014-03-27

**Authors:** Josephine H. Li, Scott V. Joy, Susanne B. Haga, Lori A. Orlando, William E. Kraus, Geoffrey S. Ginsburg, Deepak Voora

**Affiliations:** 1 Center for Personalized and Precision Medicine, Duke University Medical Center, Durham, NC 27708, USA; E-Mails: josephine.li@dm.duke.edu (J.H.L.); susanne.haga@duke.edu (S.B.H.); geoffrey.ginsburg@duke.edu (G.S.G.); 2 Division of General Internal Medicine, Department of Medicine, University of Colorado Denver Anschutz Medical Campus, Aurora, CO 80045, USA; E-Mail: scott.joy@ucdenver.edu; 3 Division of General Internal Medicine, Department of Medicine, Duke University Medical Center, Durham, NC 27710, USA; E-Mail: lori.orlando@dm.duke.edu; 4 Division of Cardiovascular Medicine, Department of Medicine, Duke University Medical Center, Durham, NC 27710, USA; E-Mail: william.kraus@dm.duke.edu

**Keywords:** pharmacogenetics, personalized medicine, medication adherence, risk assessment, health behavior, hyperlipidemia

## Abstract

Statin adherence is often limited by side effects. The SLCO1B1*5 variant is a risk factor for statin side effects and exhibits statin-specific effects: highest with simvastatin/atorvastatin and lowest with pravastatin/rosuvastatin. The effects of SLCO1B1*5 genotype guided statin therapy (GGST) are unknown. Primary care patients (n = 58) who were nonadherent to statins and their providers received SLCO1B1*5 genotyping and guided recommendations via the electronic medical record (EMR). The primary outcome was the change in Beliefs about Medications Questionnaire, which measured patients’ perceived needs for statins and concerns about adverse effects, measured before and after SLCO1B1*5 results. Concurrent controls (n = 59) were identified through the EMR to compare secondary outcomes: new statin prescriptions, statin utilization, and change in LDL-cholesterol (LDL-c). GGST patients had trends (*p* = 0.2) towards improved statin necessity and concerns. The largest changes were the “need for statin to prevent sickness” (*p* < 0.001) and “concern for statin to disrupt life” (*p* = 0.006). GGST patients had more statin prescriptions (*p* < 0.001), higher statin use (*p* < 0.001), and greater decrease in LDL-c (*p* = 0.059) during follow-up. EMR delivery of SLCO1B1*5 results and recommendations is feasible in the primary care setting. This novel intervention may improve patients’ perceptions of statins and physician behaviors that promote higher statin adherence and lower LDL-c.

## 1. Introduction

Statins are commonly prescribed to lower low-density lipoprotein cholesterol (LDL-c) and to prevent cardiovascular disease (CVD) [1]. Despite their proven efficacy and safety, long-term compliance with statin therapy is a challenge in the clinical setting. In patients with coronary artery disease (CAD), up to 50% are nonadherent to statins after one year [2,3]. The consequences of statin nonadherence are higher LDL-c and costs [4] due to higher cardiovascular mortality, hospitalizations, and revascularization procedures [3]. 

Medication nonadherence is a complex problem caused by patient, provider, and health system-based obstacles [5]. A major patient-oriented cause of statin nonadherence is statin-induced myopathy, which ranges from the rare condition of rhabdomyolysis to the more common condition of creatine kinase (CK)-negative myalgias [6,7,8]. Patient concerns about or prior, personal experiences with statin-induced side effects are a primary reason for statin nonadherence [6,7,9,10]. Provider concerns about the possibility of statin-related side effects often limit the re-initiation of statins in patients with prior side effects [11,12]. Despite these obstacles, studies show that patients with prior side effects who have discontinued statin therapy can often be rechallenged with statins and are able to tolerate them, suggesting that patient and/or provider factors surrounding side effects are transient and/or modifiable [13].

We and others have established that the *5 variant (rs4149056, defined by the minor C allele) in the hepatic transporter protein SLCO1B1 is a risk factor for statin-induced side effects and premature drug discontinuation [14,15,16]. The risk of myopathy conferred by the *5 variant appears to be statin-specific: highest with simvastatin and atorvastatin and lowest with pravastatin and rosuvastatin [15,17,18]. Given the relatively high prevalence of the *5 variant (25% of Caucasians are carriers) and its statin-specific association with statin-induced myopathy, *5 genotyping may have clinical utility by helping to inform statin selection and impact adherence. To test the hypothesis that knowledge of personalized risk of statin-induced myopathy may improve perceptions and adherence to statins, we conducted a pilot study (NCT01894217) of primary care patients who have a history of statin nonadherence. The primary outcome was the change in patients’ perceptions of their needs and concerns for statins. Additionally, we hypothesized that genotype guided statin therapy (GGST) would be associated with improved statin adherence, statin prescribing behavior, and LDL-c, compared to concurrent control subjects receiving standard-of-care. 

## 2. Results and Discussion

### 2.1. Results

**Figure 1:**
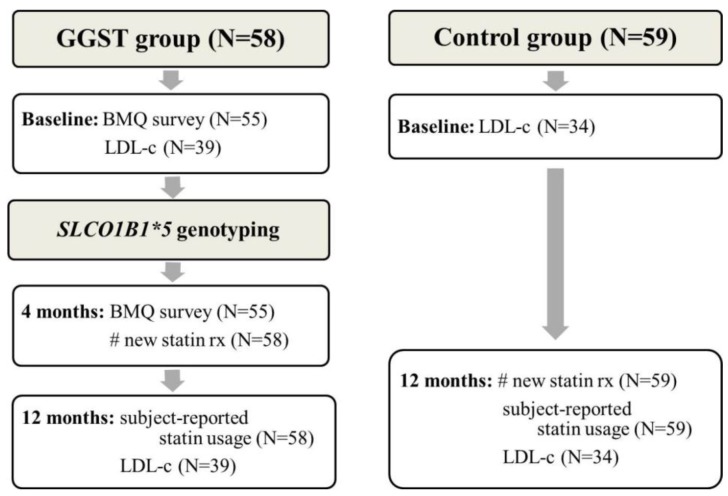
Flow diagram illustrating study design and outcome measures for individuals in the GGST and control groups. GGST = genotype guided statin therapy; BMQ = Beliefs about Medications Questionnaire; LDL-c = low-density lipoprotein cholesterol.

Figure 1 illustrates the study design and the number of patients included in each analysis. We recruited 62 individuals in the GGST arm and 59 concurrent controls. Four individuals in the GGST arm were not genotyped because they were unable to travel to clinic for the blood draw and were considered lost to follow-up. Genotypes were successfully determined in the remaining 58 GGST subjects; we identified 11 heterozygous carriers and one homozygous carrier. Due to the small number of CC individuals, we combined them with CT individuals. The frequency of the C allele in Caucasians was not different than that reported in the literature (14% *versus* 15%, *p* = 0.94). 

A description of the two cohorts is shown in Table 1. There were no statistically-significant differences between the two groups except in baseline LDL-c, where GGST subjects had a higher mean LDL-c. To take these differences into account, we subsequently adjusted all our analyses for the proportion of patients who were (or were not) at their National Cholesterol Education Program (NCEP)-defined goals. These guidelines are utilized by clinicians in initiating statin therapy and titrating therapy to the appropriate LDL-c goals for patients based on their risk factors [19].

**Table 1:** Patient characteristics in genotype guided statin therapy (GGST) and control groups.

Characteristic	GGST (N = 58)	Control (N = 59)	p*
**Age (SD)**	63.6 (9.0)	63.6 (13.3)	0.98
**Sex, male (%)**	21 (36.2)	14 (23.7)	0.16
**Race, white (%)**	46 (79.3)	38 (64.4)	0.10
**BMI (SD)**	30.4 (5.3)	32.2 (9.7)	0.22
**History of smoking (%)**	11 (19.0)	16 (27.1)	0.38
**Number of medical comorbidities (SD)**	3.8 (1.6)	3.6 (1.2)	0.41
**Total cholesterol, mg/dL (SD), N = 98**	217.3 (57.0)	198.2 (42.6)	0.06
**HDL-c, mg/dL (SD), N = 98**	51.0 (12.7)	49.9 (13.4)	0.69
**LDL-c, mg/dL (SD), N = 99**	146.4 (51.9)	122.8 (35.6)	**0.009**
**NCEP goal at baseline (%), N = 99**	16 (44.4)	20 (55.6)	0.25
**Clinic, Pickett Road (%)**	54 (93.1)	57 (96.6)	0.44

* All statistical tests compared the GGST group to the control group. Two-sided *t*-test was used for continuous variables and Fisher’s exact test for categorical variables. GGST = genotype guided statin therapy; BMI = body mass index; HDL-c = high-density lipoprotein cholesterol; LDL-c = low-density lipoprotein cholesterol; NCEP = National Cholesterol Education Program.

#### 2.1.1. Change in the Beliefs about Medications Questionnaire (BMQ) from Baseline to Four Months

Of the 58 individuals enrolled in the study, 55 completed both the baseline and four-month BMQ (Figure 1). GGST patients expressed a trend toward higher “necessity” (pre: 15.1 ± 3.8 *versus* post: 15.6 ± 4.0 *p* = 0.24) and lower “concern” (pre: 15.2 ± 4.2 *versus* post: 14.7 ± 4.3, *p* = 0.24) at four months compared to baseline (Table 2). When examining the individual components of the necessity and concerns domains, the largest changes in the BMQ were in the “need for statin to prevent sickness” (pre: 2.9 ± 0.9 *versus* post: 3.3 ± 0.9, *p* < 0.001) and the “concern for statin to disrupt life” (pre: 3.2 ± 1.4 *versus* post: 2.8 ± 1.2, *p* = 0.006). The mean necessity-concerns difference (where a higher difference is associated with higher adherence [20,21,22]) demonstrated a trend towards improvement (pre: −0.2 ± 6.3 *versus* post: 0.9 ± 6.8, *p* = 0.12). 

#### 2.1.2. Secondary Outcomes

**Figure 2:**
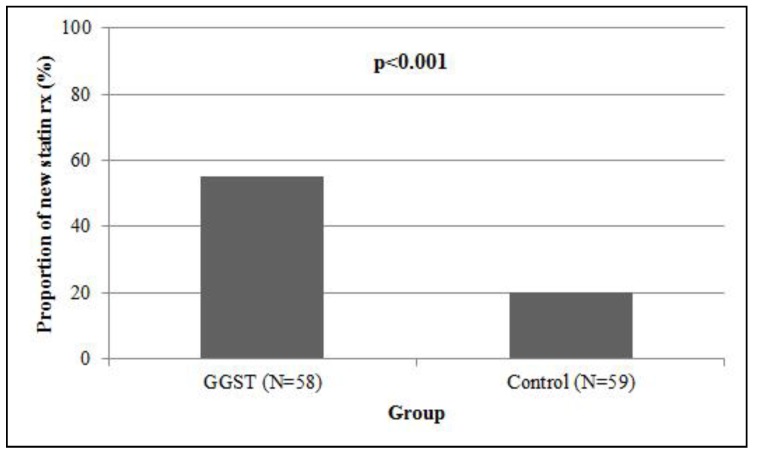
Association of GGST with new statin prescriptions. Individuals receiving GGST had a higher proportion (55%) at four months following genotyping compared to control subjects (20%) followed for one year. GGST = genotype guided statin therapy.

**Figure 3:**
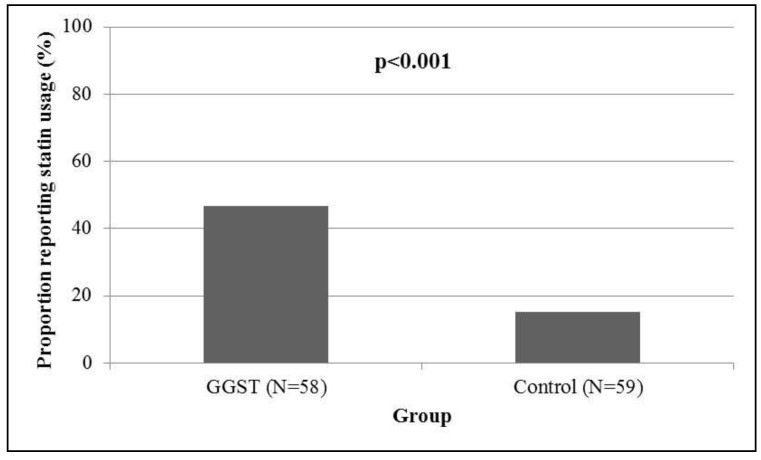
Association of GGST with subject-reported statin utilization. Individuals receiving GGST had a higher subject-reported statin utilization (47%) following genotyping compared to control subjects (15%) over a one-year period. GGST = genotype guided statin therapy.

**Figure 4:**
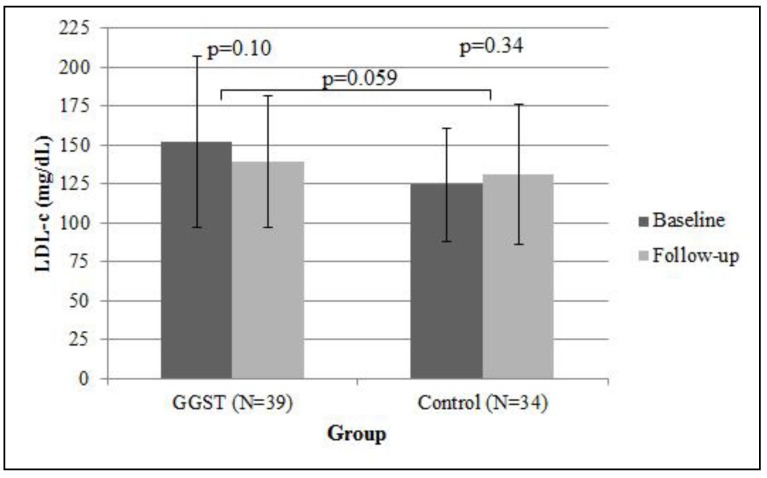
Association of GGST with change in LDL-c. Displayed are means and standard error bars of LDL-c. Individuals receiving GGST had a greater decrease in LDL-c (−12.4 ± 45.4 mg/dL) compared to control subjects (6.3 ± 37.8) over one-year follow-up. GGST = genotype guided statin therapy; LDL-c = low-density lipoprotein cholesterol.

Compared to control subjects who were followed for one year in the same clinic, GGST subjects had a higher proportion of new statin prescriptions written by their primary care physician at four months (55% *versus* 20%, *p* < 0.001, Figure 2). We performed exploratory analysis among patients who received a new statin, looking at the proportion of prescriptions that were rosuvastatin or pravastatin. Of the nine carriers in the intervention group who received a new prescription, eight received either rosuvastatin or pravastatin (89%). Twelve of the 23 noncarriers (52%) who received a new statin prescription were restarted on either rosuvastatin/pravastatin. Within the control group, four of 12 (33%) received a new prescription of rosuvastatin/pravastatin. Compared to controls, GGST subjects had higher subject-reported statin usage (47% *versus* 15%, *p* < 0.001, Figure 3). Both associations remained statistically significant (*p* = 0.002 and *p* = 0.004, respectively) after adjusting for NCEP goals at baseline. In 39 GGST subjects with pre- and post-GGST LDL-c, we observed a 12.4 ± 45.4 mg/dL decrease over a one-year follow-up (*p* = 0.10, Figure 4). In comparison, in 34 control subjects with available LDL-c at baseline and at one-year follow-up, there was a 6.3 ± 37.8 mg/dL *increase* in LDL-c (*p* = 0.34). Compared to controls, GGST subjects had a decrease in LDL-c over the duration of follow-up (*p* = 0.059). These observations were marginally attenuated (*p* = 0.08) after adjusting for NCEP goals at baseline.

**Table 2:** Changes in the Beliefs about Medications Questionnaire after receiving GGST.

	Mean score (SD)	Mean score (SD)	*p*
	at baseline	at 4 months	
**Necessity questions**			
Lowering my cholesterol requires medications	3.7 (1.0)	3.5 (1.2)	0.18
My life would have been impossible without medications to lower my cholesterol	2.4 (0.9)	2.5 (1.0)	0.46
Without medicine to lower my cholesterol, I may have become very ill	3.0 (0.9)	3.2 (1.0)	0.14
My health depended on medicine to lower my cholesterol	3.1 (1.1)	3.2 (1.0)	0.72
My medicine to lower my cholesterol protected me from becoming sick	2.9 (0.9)	3.3 (0.9)	**<0.001**
**Concern questions**			
Having to take medicine to lower my cholesterol worried me	3.2 (1.3)	3.1 (1.2)	0.38
My medicine to lower my cholesterol was a mystery to me	2.4 (1.1)	2.4 (1.0)	0.81
My medicine to lower my cholesterol disrupted my life	3.2 (1.4)	2.8 (1.2)	**0.006**
I sometimes worried about becoming too dependent on medicine to lower my cholesterol	2.5 (1.2)	2.7 (1.2)	0.35
I sometimes worried about the long-term effects of medicine to lower my cholesterol	3.9 (1.1)	3.8 (1.1)	0.56
**Mean necessity score**	15.1 (3.8)	15.6 (4.0)	0.24
**Mean concern score**	15.2 (4.2)	14.7 (4.3)	0.24
**Mean differential** **(necessity – concern)**	−0.2 (6.3)	0.9 (6.8)	0.12

Each question was answered using a 5-point Likert scale, ranging from 1 = strongly disagree to 5 = strongly agree. Items on the Necessity and Concerns scales were individually summed (total scores ranged from 5–25, with higher scores indicating stronger beliefs). Mean scores on each scale were reported as well as the mean difference between the Necessity and Concern scores. GGST = genotype guided statin therapy.

#### 2.1.3. Stratified Analyses by *5 Carrier Status

To explore the extent to which our observations were similar in carriers *versus* noncarriers we performed stratified analyses. We found that the magnitude and direction of the change in the responses to the “need for statin to prevent sickness” and “concern for statin to disrupt life” questions were the same in both carriers and noncarriers (Table S1). There was no statistically-significant difference in the proportion of new statin prescriptions between carriers and noncarriers (carriers: 75% *versus* noncarriers: 50%) and both subgroups had a higher proportion than the control group. Carriers and noncarriers had similar subject-reported statin utilization (carriers: 50% *versus* noncarriers: 46%). Lastly, the change in LDL-c from baseline to follow-up was in the same direction in carriers and noncarriers (carriers: −23.2 ± 66.4 mg/dL *versus* noncarriers: −9.2 ± 38.0 mg/dL). 

### 2.2. Discussion

Statin nonadherence is a multifactorial problem that results in higher cholesterol, hospitalizations for cardiovascular events (e.g., myocardial infarction, revascularization, and stroke), and costs [3,4]. Provision of pharmacogenetic test results to the patient and provider has the potential to impact medication adherence, through improving patients’ knowledge of their disease, reducing concerns about the medication, and fostering a sense of shared decision-making [23,24]. Based on these principals, we tested the feasibility of a novel, innovative approach to improve statin adherence that is tailored to an individual’s SLCO1B1*5 genotype and addresses a major driver of statin adherence in the primary care population. 

We acknowledged that statin adherence requires both patient and provider behaviors in a sequence of steps, which include the physician discussing risks and benefits of the medication and writing a prescription, the patient agreeing to take a statin, the patient filling the prescription at the pharmacy, the patient taking the prescription and receiving the LDL-c lowering benefits of the drug, and finally sustaining these efforts over a long period of time with the intent to reduce cardiac morbidity and mortality. Although we did not measure long-term clinical events, our study demonstrated consistent findings across this continuum of events that overall contribute to improved statin adherence. For example, the trend towards improved necessity and reduced concern for statins on some elements of the BMQ are associated with higher levels of statin adherence [20,21]. The observed changes in the BMQ reinforce the concept that patients’ beliefs about their medications are modifiable characteristics. 

Although we informed carriers of their heightened risk for side effects, we mitigated the perception of increased risk by providing recommendations for alternative strategies. We observed that individuals who underwent genotyping had a higher proportion of new statin prescriptions, which was likely a result of the patients’ increased willingness to restart a statin and/or reassurance by the provider. This finding is remarkable because our control population was seen by the same providers over the same time period as the intervention and had similar inclusion/exclusion criteria. Since indications for statin therapy are long-term indications, we expected that the controls would have had ongoing reinforcement for the need for statins. The higher proportion of new prescriptions in the GGST group suggested that provision of genetic information can result in changes in physician prescribing behavior since physicians may be reluctant to restart a statin in patients who have been nonadherent perhaps due to side effects. In our exploratory analysis, we calculated the proportion of new statin prescriptions that were either rosuvastatin or pravastatin as another measure of provider behavior. Because our messages delivered through the electronic medical record (EMR) recommended these two statins in SLCO1B1*5 carriers, we would anticipate that providing genotyping would alter physician prescribing patterns toward prescribing more rosuvastatin and pravastatin. While our sample sizes were small, we did observe the highest proportion of rosuvastatin/pravastatin prescriptions in carriers compared to control subjects who were not genotyped. Interestingly, noncarriers also had a higher proportion of rosuvastatin/pravastatin prescriptions compared to controls, though not as pronounced as that seen in carriers, suggesting that there was still some effect of GGST on physician prescribing behavior regardless of test results. We also observed that the GGST group had higher subject-reported statin utilization, suggesting that subjects filled their new prescription and took the medication consistently enough to report it in subsequent clinic visits. Finally, GGST subjects had a decrease in LDL-c compared to an increase in LDL-c in the control group, suggesting that GGST subjects were taking enough statin to receive the LDL-c lowering benefits. 

Our stratified analyses showed that the effect of genotyping was not limited to *5 carriers. Our explanation for the consistent effects across both genotypes was that not only did carriers receive a benefit from tailored statin therapy, but also noncarriers received a positive message about their lower risk of side effects. We speculate that the positive message in noncarriers was reassuring to patients and providers, enabling new prescriptions and higher adherence. By providing information about patients’ genetic risk of side effects, we introduced a new variable into the conversation between patients and providers to change their perceptions of statins in a direction that is associated with improved adherence. Furthermore, genetic testing may have offered a new avenue for shared decision-making in the patient-provider relationship and for alleviating patient concerns.

Since there is no gold-standard measurement of medication adherence, a combination of both direct and indirect methods is often employed [25]. Most existing studies on interventions to improve statin adherence utilize pharmacy claims data, which we did not have access to for our study. Instead, we used several indirect measures to quantify adherence such as questionnaires, physiological markers such as LDL-c, and physician prescription rates of statins. While these measures have their limitations, we felt that they were less burdensome and costly. 

One recent randomized study in the outpatient setting of 201 patients with indications for statins either for primary or secondary prevention examined the impact of nurse-led cardiovascular risk-factor counseling on statin adherence, lipid levels, and BMQ scores, which are indirect measures of statin adherence similar to those we explored [26]. The study investigators found that self-reported medication adherence (measured as the percentage of statin taken within the last month) was higher in the intervention group compared to the control (95%–100% *versus* 90%–95%, *p* < 0.05). Though our findings in regards to self-reported statin utilization were in the same direction with respect to the impact of *5 genotyping, the rates of statin utilization were much lower overall. This difference in utilization can be explained by our inclusion criteria; while the Nieuwkerk *et al.* study examined patients who were statin naïve, we targeted those who had prior difficulties with statins and at higher risk for future nonadherence. Nieuwkerk *et al.* [26] observed that in a subset of individuals receiving statins for primary prevention, LDL-c levels decreased in both groups (intervention: from 186 ± 46 mg/dL to 103 ± 3.9 mg/dL, control: from 189 ± 54 mg/dL to 116 ± 3.9 mg/dL). In our study, we observed a *decrease* in LDL-c and perhaps a small *increase* in controls, which suggest that our intervention had greater relative lowering compared to other prior interventions.

Nieuwkerk *et al.* [26] also measured the BMQ during follow-up and observed a decrease in mean concerns score of 1.8 in the intervention group compared to the control (13.0 ± 0.4 *versus* 14.8 ± 0.4, *p* < 0.01). In our study, we observed a smaller non-significant decrease of 0.5 in the mean concerns score from baseline to four-month follow-up. However, our intervention of SLCOB1*5 genotyping and providing guided recommendations via the EMR is an easier intervention to implement compared to a nurse-led intervention. We would anticipate that combining our genotype-guided intervention with cardiovascular risk-factor counseling may lead to a larger drop in the mean concerns score.

Additionally, our work was consistent with the AKROBATS trial, where kinesin-like protein 6 (*KIF6*) genetic testing was associated with improved statin adherence in a non-randomized study [27]. Carriers of a *KIF6* polymorphism are at a higher risk for cardiovascular events and were thought to receive a greater benefit from intensive statin therapy [28,29]. Although several negative studies have been published that question the original observations surrounding *KIF6* [30,31,32], patients in the AKROBATS trial did not have this information and responded to the *KIF6* results provided to them in a positive manner [27]. Similar to the AKROBATS trial, which delivered test results through the mail, our study mimicked the processes by which providers handle laboratory information and how patients receive test results. As a consequence, we minimized disruptions in clinic work flow that are often barriers to genetic testing in the primary care setting. 

#### 2.2.1. Limitations

The largest limitation to our study was the lack of randomization. There is a possibility that the changes we observed in patient behavior, in part, may have been driven by closer physician follow-up in the intervention group or simply the act of counseling a patient, rather than the information gleaned from *5 genotyping. We minimized differences between control and GGST subjects by selecting controls from the same clinics and from the same time period but we observed that GGST individuals had a higher baseline LDL-c. This could have been a result of physicians referring patients who had higher LDL-c for genotyping. However, we found no statistically-significant differences between the proportion of individuals at their NCEP goals at baseline between the GGST and control groups, with the majority of individuals not at goal. We did observe fewer females in the intervention group compared to the control group. This may have resulted in an overestimation of the benefits of genotyping since female sex is associated with statin-induced side effects, which can lead to nonadherence [14,15]. However, when we additionally adjusted for sex in our multivariable analysis, we found that our results did not change (*p* < 0.05 for new statin prescription and for statin utilization, *p* = 0.09 for LDL-c). Furthermore, the use of a concurrent control group may have overestimated the true effect of GGST because patients who chose to participate in the study may be naturally more proactive about their own health. Lastly, we did not have pharmacy level data available and could not confirm patient adherence at the level of prescription refill data. To address all of these limitations, we are directly testing *5 genotyping in a larger randomized control trial that is currently underway (NCT01894230). Our trial will randomize patients to receive *5 genotyping plus usual care *versus* usual care only in order to further illustrate the impact of providing genotype information on patient and provider behaviors.

Despite the small sample size of our study, we demonstrated that delivery of a novel intervention whereby statin therapy is tailored to *5 genotype is feasible and that genetic test results can be successfully integrated into an EMR and delivered within the clinical workflow of a primary care setting. This intervention may improve patients’ perceptions of statins and was associated with physician and patient behaviors that promoted higher statin adherence and lower LDL-c. Our pilot study laid the foundation for a large, ongoing randomized control trial that compares the benefits of genotype guided statin therapy against non-guided therapy.

## 3. Experimental

### 3.1. Study Population

Patients were recruited from Duke Primary Care at Pickett Road (DPC) and Duke Center for Living (CFL). Patients were eligible if they (1) were seen in either clinic within the last year, (2) had an indication for statin therapy (e.g., diagnosis of diabetes, CAD, or hyperlipidemia) determined by electronic chart review, (3) were nonadherent (defined as not currently taking prescribed statin therapy) to statins as determined by electronic chart review or confirmed by the primary care provider, and (4) had access to Duke Healthview Portal, a web-based application that enables patients to receive test results and communicate with their health care providers. Patients were excluded if they (1) had prior rhabdomyolysis with statins, (2) had an unexplained elevation in liver enzymes >3 times the upper limit of normal, or (3) were receiving medications that interfered with statin metabolism. 

Concurrent controls were defined as individuals receiving care in same clinics during the same time as GGST subjects. Potential controls were identified through the Duke Enterprise Data Unified Content Explorer (DEDUCE), a web-based query tool of billing, laboratory, prescription, and subject-reported medication use data from patients in the Duke University Health System (DUHS) [33]. Individuals were included if they (1) previously reported statin utilization, (2) had not reported taking a statin for at least 3 months for any reason, (3) had a primary care clinic visit within the last year, and (4) another visit in the subsequent year. Exclusion criteria were the same as in the GGST group, and eligibility criteria were verified by electronic chart review. Data for comparison of secondary outcomes for concurrent controls was collected through the EMR via a waiver of informed consent. This study was approved by the Duke University Institutional Review Board. The study was registered with clinicaltrials.gov (NIH trial registry number: NCT01894217) [34]. 

### 3.2. Study Intervention

Eligible patients for GGST who provided informed consent were provided genotyping for SLCO1B1*5 in the Duke Molecular Diagnostics Laboratory. Genotyping results were simultaneously sent to the provider through EMR and to the patient through Healthview. The test report included genotype-specific information about the patient’s risk of side effects on statin therapy and recommendations for the provider to consider when prescribing a new statin. Briefly, *5 carriers and their providers were informed that they were at greater risk for side effects when taking simvastatin or atorvastatin and therapy with pravastatin or rosuvastatin was recommended (Table S2). Noncarriers and their providers were notified that they were at lower risk and could restart any statin that had not caused side effects in the past. Study enrollment occurred from October 6, 2011, to August 1, 2012, and follow-up for GGST subjects began following receipt of test results. For controls, follow-up began on October 6, 2011, and continued for one year.

### 3.3. Study Definitions and Outcomes

#### 3.3.1. Patient characteristics

Baseline variables were collected through case report forms (CRFs) and included age, sex, race (coded dichotomously as white or nonwhite), body mass index (BMI), and the following medical comorbidities: myocardial infarction, hypertension, hyperlipidemia, stroke, diabetes, congestive heart failure, arthritis, asthma, hematological disorder, chronic obstructive pulmonary disease, dementia, osteoporosis, obesity, seizure, thyroid disease, epilepsy, glaucoma, and depression. Smoking history was collected using DEDUCE. Baseline high-density lipoprotein cholesterol (HDL-c) and LDL-c, defined as within one year prior to genotyping, were gathered from DEDUCE. If multiple lipid levels were available, the measurement with the latest report date within that time period was used. The proportion of individuals who reached their NCEP goals at baseline was calculated [19].

#### 3.3.2. Beliefs about Medications Questionnaire (BMQ)

The primary outcome was the change in the BMQ, a validated tool that assesses patients’ perceived necessity for their prescribed medication and their concerns about adverse effects of the medication [20,21]. We modified the BMQ to report on statin therapy and administered the survey prior to and at 4 months after genetic testing. Each question was answered on a 5-point Likert scale from 1 = strongly disagree to 5 = strongly agree. Total scores for the Necessity and Concerns scales ranged from 5 to 25 (higher scores indicated stronger beliefs). Higher necessity, lower concern, and higher necessity-concerns difference scores have been found to be significant predictors of greater medication adherence [20,21,22].

#### 3.3.3. Secondary Outcomes

In GGST subjects, the proportion with new statin prescriptions written by their primary care providers was measured at four months following genotyping. We ensured that the control population had equivalent access to their physicians by choosing a longer, one-year follow-up period to allow for new prescriptions to be written. In GGST and control subjects, we collected subject-reported statin utilization generated from medication reports collected at the time of clinic visits. During routine clinic visits at DPC or CFL, the intake nurse asks each patient about their current medications, verifies medication dosages and frequency, and records any changes to medications in the EMR. As a result of this face-to-face interaction between the nurse and the patient, a patient medication reconciliation report is generated for each patient encounter. We gathered the latest three reports at the end of one-year follow-up. Statin adherence was a dichotomous outcome defined as subjects having a statin on their medication report for all three reports. LDL-c at one-year follow-up for GGST and control subjects were obtained in the same manner as baseline LDL-c (described above) and were used to calculate the change in LDL-c over one year.

### 3.4. Statistical Analysis

Hardy-Weinberg Equilibrium for *5 was checked in Caucasians within the GGST group. Differences in baseline characteristics between GGST and control subjects were assessed using a two-sided *t*-test for continuous variables and Fisher’s exact test for categorical variables. For all variables that did not approximate a normal distribution, non-parametric tests were utilized and did not alter the conclusions. A paired t-test assessed changes from baseline to four months in the BMQ. Logistic regression tested the association between GGST and the proportion of new statin prescriptions and subject-reported statin usage. To compare the change in LDL-c from baseline to one-year follow-up in the GGST and control groups, a paired *t*-test was used. To assess the association of GGST on the change in LDL-c from baseline to one-year follow-up, analysis of variance (ANOVA) was used. Multivariable adjustment was performed for secondary outcomes, adjusting for any differences in baseline characteristics between the GGST and control groups. Because clinicians rarely interpret LDL-c in isolation and instead utilize the patients’ NCEP-defined LDL-c goal to initiate statin therapy, we adjusted for the proportion of individuals at NCEP goal at baseline. Statistical analyses were performed using R statistical software [35]. 

## 4. Conclusions

Delivery of SLCO1B1*5 results and recommendations is feasible via the EMR in the primary care setting. This novel intervention may improve patients’ perceptions of statins and physician behaviors that promote higher statin adherence and lower LDL-c. 

## Supplementary Files

Supplementary File 1 Supplementary Material (PDF, 265 KB)
