# Supplementary material for: Genetically Guided Statin Therapy on Statin Perceptions, Adherence, and Cholesterol Lowering: A Pilot Implementation Study in Primary Care Patients
Source: J Pers Med. 2014 Mar 27;4(2):147–62. doi: 10.3390/jpm4020147 (PMC4263970; doi:10.3390/jpm4020147)
Supplement: Supplementary File 1 — Supplementary Material (PDF, 265 KB) [file jpm-04-00147-s001.pdf]

Supplementary Material

Supplemental Table S1. Changes in selected items from the Beliefs about Medications Questionnaire after receiving GGST, stratified by **5* carrier status.

	Group	Mean score (SD) at baseline	Mean score (SD) at 4 months	*p*
My medicine to lower my cholesterol protected me from becoming sick	All (N = 55)	2.9 (0.9)	3.3 (0.9)	**<0.001**
	Carriers (N = 12)	3.0 (0.6)	3.3 (0.7)	
	Noncarriers (N = 43)	2.8 (1.0)	3.3 (0.9)	
My medicine to lower my cholesterol disrupted my life	All (N = 55)	3.2 (1.4)	2.8 (1.2)	**0.006**
	Carriers (N = 12)	2.8 (1.5)	2.4 (0.9)	
	Noncarriers (N = 43)	3.3 (1.6)	2.9 (1.3)	

Each question was answered using a 5-point Likert scale, ranging from 1 = strongly disagree to 5 = strongly agree. GGST = genotype guided statin therapy.

Supplemental Table S2. Genotype-specific interpretation and instructions provided to physicians through the electronic medical record and to patients through Healthview based on **5* carrier status.

	Genotype group	
	**Carrier of **5***	**Noncarrier of **5***
Information provided to providers	Your patient carries at least one copy of the SLCO1B1*5 genetic variant. This puts him/her at increased risk for rhabdomyolysis (~4.5 fold) and/or CK-negative myopathy (~2 fold), particularly when taking simvastatin or atorvastatin as compared to pravastatin or rosuvastatin. If statin therapy is needed, consider using pravastatin or rosuvastatin as first line therapies with doses as low as possible. Alternate day dosing is also recommended.	Your patient does not carry the SLCO1B1*5 genetic variant. This puts him/her at very low (less than 5 in 1000) risk for rhabdomyolysis and/or CK-negative myopathy. Consider restarting any statin that has not caused side effects in the past.
Information provided to subjects	A genetic test was performed to determine if you are at increased risk to develop muscle pains while taking statin medications. Your genetic test results show that you are at increased risk to develop muscle pains while taking certain statin medications. In research studies, patients who have this genetic test result are more likely to have muscle pains when taking Zocor (simvastatin) or Lipitor (atorvastatin). Because you are at increased risk, your physician has provided a different prescription for you. By choosing this new medication for you – either Pravachol (pravastatin) or Crestor (rosuvastatin) – your physician hopes to reduce your cholesterol and to prevent heart disease, but with a lower risk of developing muscle pains	A genetic test was performed to determine if you are at increased risk to develop muscle pains while taking statin medications. Your genetic test results show that you are not at increased risk of developing muscle pains while taking statin medications. The rest of the population has a genetic test result that increases their chances of having muscle pains when taking Zocor (simvastatin) or Lipitor (atorvastatin). Because your risk of severe side effects like muscle pain is less than 5 in 1000 or 0.05%, your physician has provided a new prescription for you. By choosing the best medication for you, your physician hopes to reduce your cholesterol and to prevent heart disease with fewer side effects.
